# Experimental study on strength and failure characteristics of sandstone rock mass with complex cataclastic structure using 3D printing models

**DOI:** 10.1038/s41598-023-31957-2

**Published:** 2023-03-25

**Authors:** Shan Dong, Zhichun Lu, Xi Hu

**Affiliations:** 1 Badong National Observation and Research Station of Geohazards, China University of Geosciences, Wuhan, 430074 China; 2 Three Gorges Research Center for Geohazards, China University of Geosciences, Wuhan, 430074 China; 3 CISPDR Corporation, Wuhan, 430074 China

**Keywords:** Geology, Engineering

## Abstract

A cataclastic rock mass is a poor type of engineering geological rock mass. The determination of the shear failure characteristics and shear strengths of cataclastic rock masses can provide key basis for the design and construction of infrastructure. Physical model samples of a sandstone cataclastic rock mass were first produced by a combination of three-dimensional (3D) printing technology and manual pouring. Shear tests were conducted with respect to the shear stresses parallel to the trace line plane and perpendicular to the trace line plane of the cataclastic rock mass model. Based on an extensive analysis of the shear failure characteristic, shear stress evolution characteristic curve and shear strength. When the shear stress was parallel to the trace line plane, and when the rock block that was cut and confined by the trace line exhibited a significant tip, the end stress concentration effect of the cataclastic rock mass was more significant during the shear process with the anisotropy of the rock block increased. In addition, the shapes of the rock blocks that were confined and cut by the joints were the main influencing factors of the strength of the cataclastic rock mass. When the shear stress was perpendicular to the trace line plane, the structure of the rock wall was the main influencing factor of the deformation and failure process of the shear failure plane and the shear strength. The physical and mechanical properties of the shear failure plane of the cataclastic rock mass were found to be closely related to the joint–rock wall system characteristics of the cataclastic rock mass. Therefore, when determining the shear strength of cataclastic rock mass, the shape and combination form of the rock block, shear direction, and structural failure characteristics of the rock wall should be comprehensively considered during the shear process.

## Introduction

A cataclastic rock mass is a poorer type of engineering rock mass, which is extensively developed in large active fault zones, deep canyons, and regions with complex lithological structures^1,2^. Due to the high heterogeneity, discontinuity, and anisotropy of a cataclastic rock mass, its integrity and overall strength are low, in addition to its stability, which is the focus of large-scale engineering construction. On the one hand, when the cataclastic rock mass is exposed, with the release of the confining stress, the rock block rebounds into a state of relaxation, and it is difficult to obtain the original sample that represents the characteristics of the actual cataclastic rock mass. On the other hand, the spatial distribution of joints of the cataclastic rock mass typically exhibits a certain randomness, and the irregular extension and intersection of the joints constitute the unique network structure of the rock mass, which determines various engineering properties of the rock mass. This increases the complexity of the analysis conducted on the shear failure characteristics of cataclastic rock masses. Numerous studies revealed that the analysis of the shear failure characteristics and shear strength of cataclastic rock masses can provide a critical technical basis for the design, construction and normal safe operation of hydropower, transportation, underground engineering, oil, and gas pipelines, in addition to mines.

As a limitation to the study of the physical and mechanical properties of cataclastic rock masses, it is challenging to repeatedly produce a cataclastic rock mass sample with a consistent joint network model. In recent years, three-dimensional (3D) printing technology has provided an effective method for the physical and mechanical experimental research and comprehensive analysis of the corresponding mechanism for natural complex rock masses^3,4^. Xiong et al.^5^ prepared natural joints of rock mass based on 3D scanning and printing techniques, and conducted experimental verification. Fereshtenejad and Song^6^ evaluated the shear strength of a rock mass containing non-persistent joints using 3D printed and plaster specimens. Wang et al.^7^ used a 3D printer to create a batch of rock masses with discontinuous joints, and determined the failure strength of rock masses with different joints based on uniaxial compression tests and direct shear tests. Huang et al.^8^ prepared an irregular columnar joint network model by combining a Voronoi diagram stochastic simulation and 3D printing technology. Feng et al.^9^ used 3D printing technology to print rock mass samples with different combinations of joints, which were then subjected to shear tests. Although 3D printing technology has been able to realize the production of model samples of various materials, most of the similar materials used to produce rock samples at this stage are based on plain concrete^10–12^. Due to the rapid solidification of plain concrete, it is easy to block the nozzle of 3D printer, so it is difficult to realize the mixed printing of plain concrete and other materials. Using 3D printing technology to make structural sample, combined with manual pouring, can simulate the hard rock of cataclastic structure to the maximum extent, and realize the model sample production of cataclastic structure rock mass.

Numerous studies were conducted on the shear properties and shear strengths of jointed rock masses based on shear stress. For example, Fan and He^13^ carried out a direct shear test on a rock mass with dense directional intermittent joints, and proposed that the failure mechanism of a rock mass with dense directional intermittent joints is compression shear tearing failure caused by a high concentration of end stress, which finally forms a stepped failure mode. Cui^14^ carried out direct shear tests on continuous planar-joints, discontinuous stepped-joints, and discontinuous open-joints; and the shear behaviour of the continuous and discontinuous joints was found to be dependent on the normal stress. Asadizadeh et al.^15^ performed direct shear tests on artificial rock specimens with two parallel (stepped and coplanar) non-persistent joints, and studied the influences of the bridge length, bridge angle, joint roughness coefficient, and normal stress on the shear strength and cracking process of non-persistent jointed rock. Yang et al.^16^ studied the influence of granite samples with discontinuous joints on the mechanical behaviour via direct shear tests, and observed the following three different failure modes at the rock bridge: (a) shear failure, (b) compression shear failure, and (c) tensile failure. Although these studies made significant contributions to the determination of the shear properties of joined rock masses, most of the tests were focused on the joints of the rock masses. In addition, there are few reports on the shear properties of the entire cataclastic rock mass.

In this study, 3D network models of the joint of a rock mass with different cataclastic structures were established based on field investigation. Thereafter, the physical model samples of a sandstone cataclastic rock mass were developed by a combination of 3D printing technology and manual pouring. By carrying out shear tests in different manners on the physical model samples of the cataclastic rock mass, the shear characteristics of the rock mass with different cataclastic degrees were comprehensively investigated. The findings of this study can serve as a theoretical basis for the investigation of the mechanical properties of cataclastic rock masses, and the disaster initiation mechanism of cataclastic rock mass slopes. Moreover, the findings can facilitate the prediction and determination of the cause of major geological disasters, which is critical to engineering applications and theoretical research.

## Materials and methods

### Model design of network models of intersecting rock mass joints

**Figure 1:**
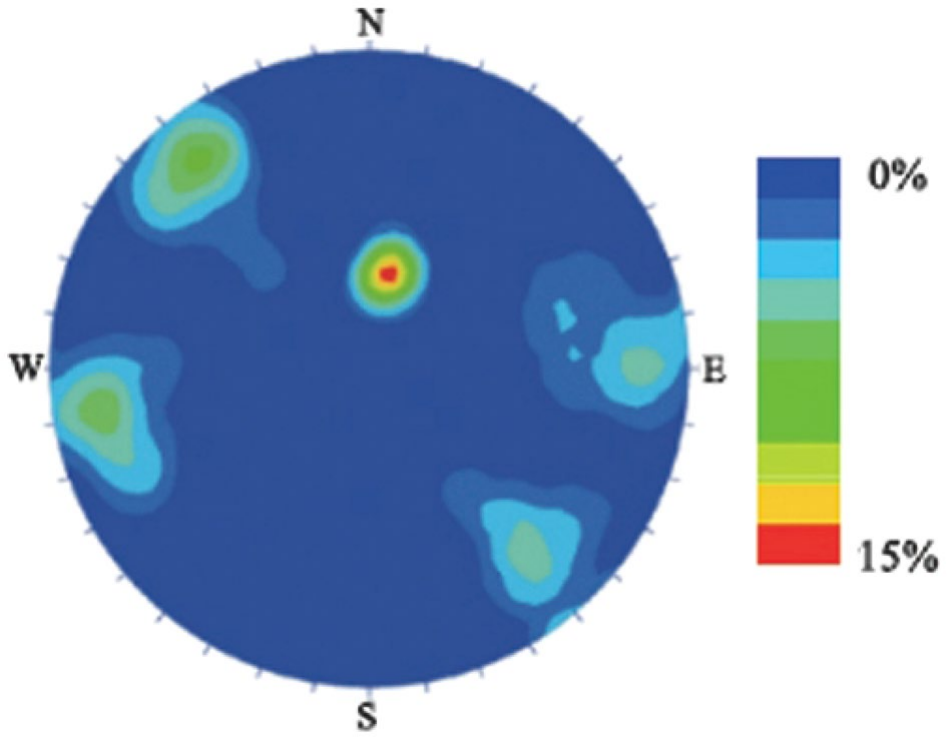
Statistical diagram of structural plane distribution of the Daguangbao landslide.

The model proposed in this paper can be mainly considered as a cataclastic rock mass, which is combined, intersected, and surrounded by two sets of intersecting joints. The Longmenshan fault zone is a common seismic zone in China^17,18^. Due to tectonic stress and superficial transformations, numerous cataclastic rock masses are formed, which are of significant representativeness and research significance^19^. Field investigations and statistics were conducted on the rock mass joints of the Daguangbao landslide in Longmenshan fault zone, and three main structural aspect groups of research point development (Fig. 1) were identified: I, 350°–15°∠ 25°–45°; II-①, 61°–100°∠ 61°–90°; II-②, 240°–280°∠ 61°–90°; III-①, 120°–150°∠ 61°–90°; III-②, 300°–326°∠ 61°–90°.

**Figure 2:**
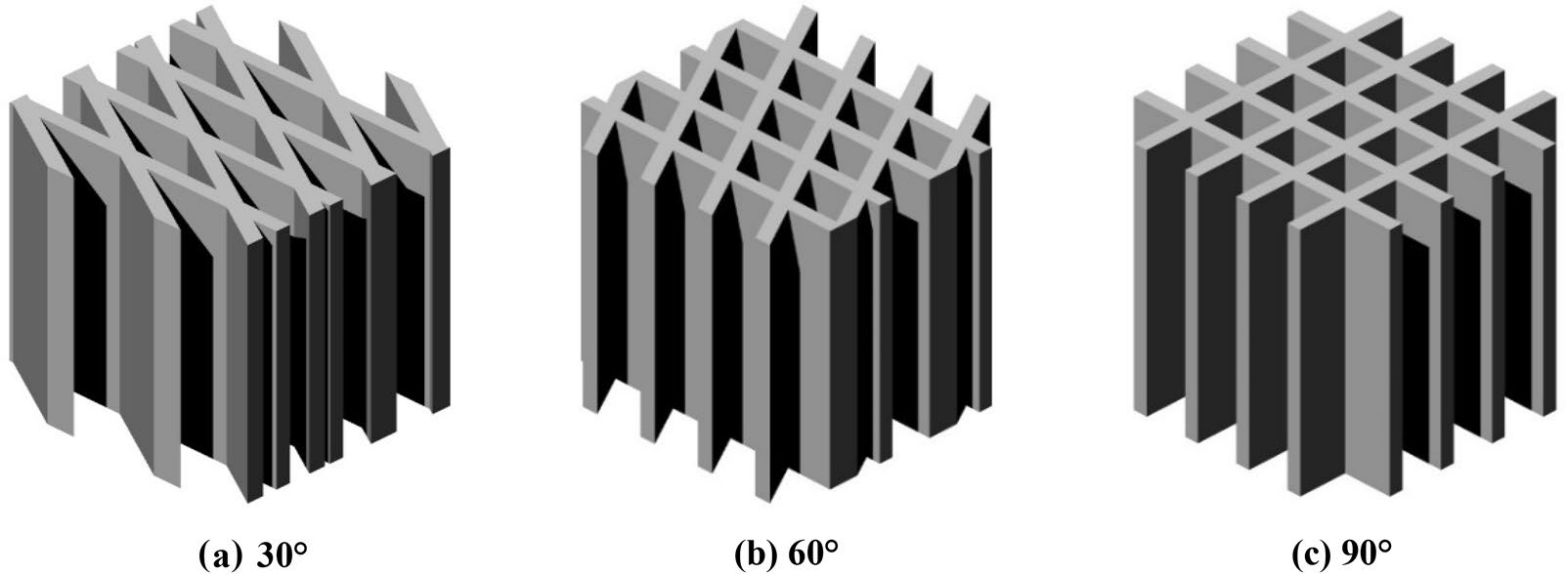
The network models of rock mass joints developed using ANSYS software.

Using the plane unit normal vector to transform the occurrence of the joints, the intersection angles of Group I and Group II joints were mainly in the range of 38.13°–58° and 58°–90°; the included angles of Group I and Group III joints mainly ranged from 21.72°–61° and 61°–90°; and the included angles of Group II and Group III joints were mainly in the range of 17.29°–64° and 64°–90°. In this study, the method of averaging was used to determine the lower critical point, middle critical point, and upper critical point of the intersection angle of the joints, which were 26°, 61°, and 90°, respectively. Furthermore, considering the representativeness of the sample and the convenience of sample preparation, the final intersection angles of the two groups of intersecting joints were 30°, 60°, and 90° respectively. Thereafter, using Analysis of Systems (ANSYS) software, network models of the rock mass joints with intersection angles of 30°, 60°, and 90° were established (Fig. 2).

### Three-dimensional printing joint network model

**Figure 3:**
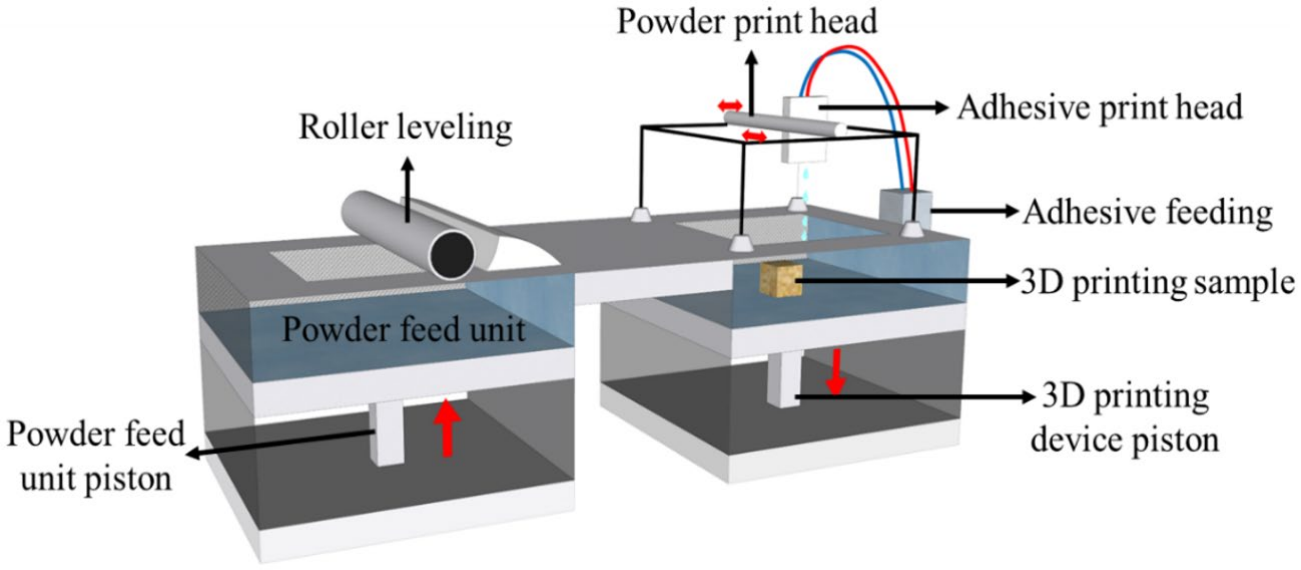
Printing mechanism of 3D printer.

In this experiment, the S-Max Pro™ 3D printer was used to print the joint network entity model. The S-Max Pro™ 3D printer, which uses an efficient print head and a full-automatic recoat machine, can print different materials and achieve a maximum printing speed of 1351/h (with a printing layer thickness of 0.26–0.38 mm). The S-Max Pro™ 3D printer uses the feeding cylinder to provide the powder particles of the printing material, spreads the printing particles in a layer on the printing work area via the printing nozzle, and then sprays a layer of adhesive on the solid part of the printed specimen to bind the material particles. When the powder particles of the previous layer are cohesive, the powder feeding piston rises by one printing layer height to start printing the subsequent layer. This process is then repeated until the solid model is printed (Fig. 3).

**Figure 4:**
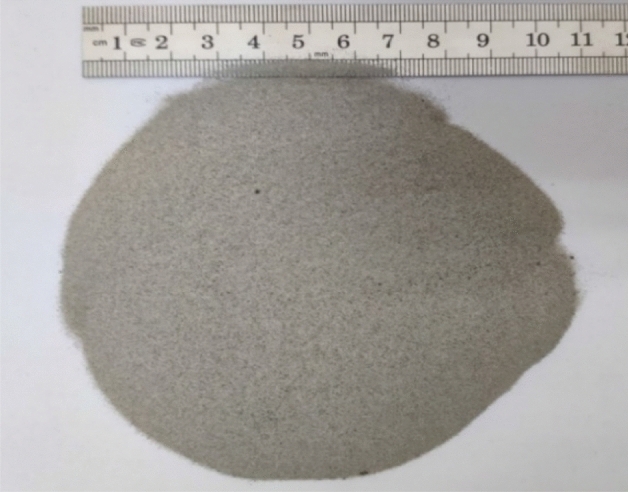
The 3D printing of sand particles.

**Figure 5:**
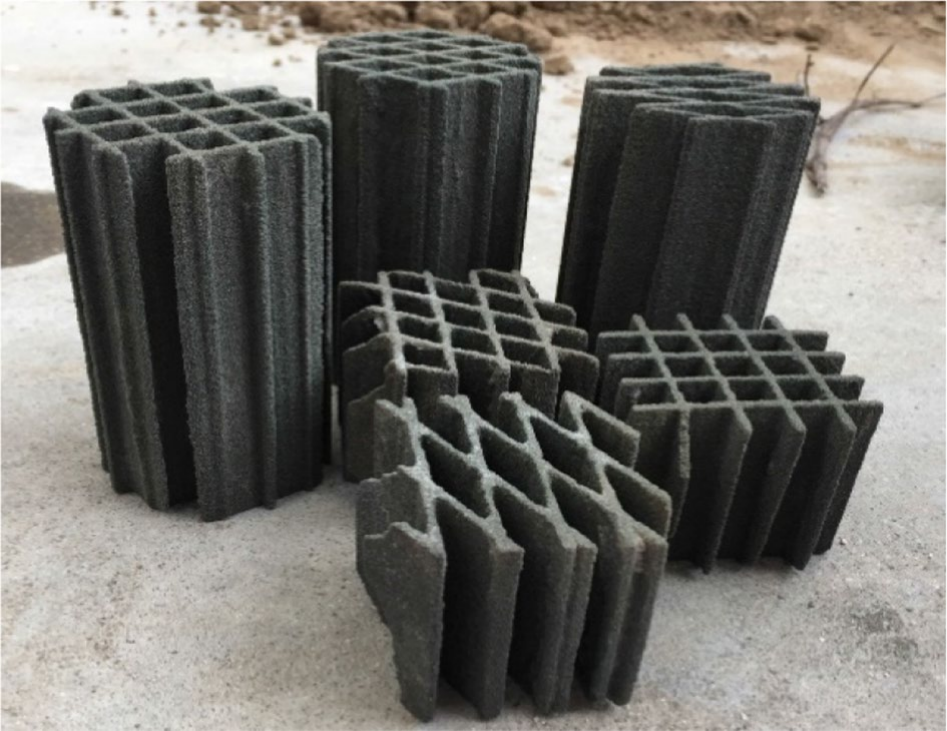
The 3D printing of the structural plane network model sample.

In this study, for the 3D printing of the plane network model of rock mass structures, artificial sandstone powder particles with particle sizes of 0.1–0.3 mm were used as aggregates (Fig. 4), which were bonded with no-bake adhesive (furan adhesives). The rock mass discontinuity network model developed using ANSYS software was imported into the S-Max Pro™ 3D printer, which automatically printed the 3D network model of the cataclastic rock mass joint (Fig. 5). Due to the extremely low strength of the printed hollow rock mass joint network model, the voids of the joint and fissure network after printing were filled with fine sand to prevent damage to the joint network model during transportation.

### Sample development of cataclastic rock mass model

#### Ratio of similar materials of rock samples

Considering that the mechanical properties of plain concrete are stable, and changing the material ratio can cause its main mechanical properties to be similar to the simulated rock properties, plain concrete was selected as a similar material in this study. Considering sandstone as the research object, by conducting shear test analysis on the intact similar material samples (no joint) and a comparison of the rock samples made with different cement concrete proportions, the plain concrete with a mass ratio of cement: fine sand: water = 2:1:1 was finally selected for pouring in this test. It should be noted that the feasibility of sample pouring was considered. When developing samples, they were simultaneously poured with applied vibration to ensure that the samples contained no bubbles until the surface was flat. Table 1 presents the fundamental mechanical parameters of the selected cement slurry pouring samples, which were relatively close to the medium hard sandstone.

**Table 1:** Mechanical parameters of medium hard sandstone and similar materials.

Material	Uniaxial compressive strength (MPa)	Cohesion (MPa)	Friction angle (°)	Elastic modulus (GPa)	Poisson’s ratio
Medium hard sandstone	53.59	3.99	49.7	37.24	0.182
Similar materials	54.67	3.93	51.8	37.79	0.176

#### Sample making

**Figure 6:**
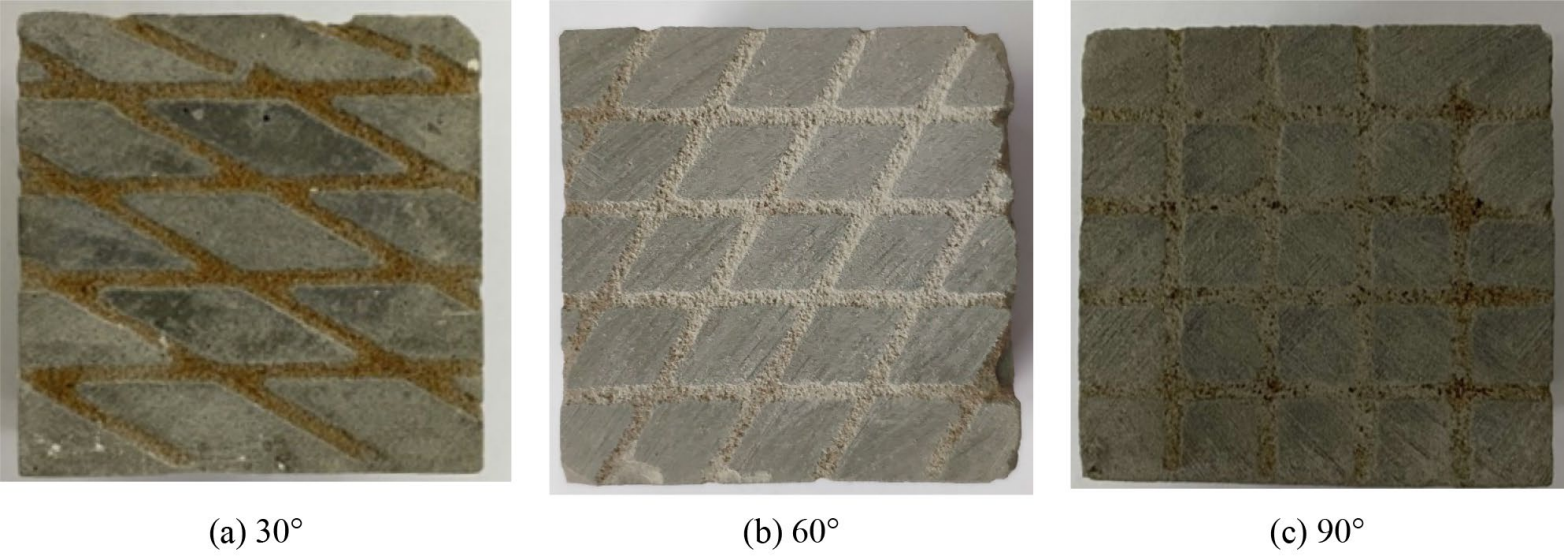
Poured cataclastic rock mass sample.

Based on the research on the joint network model and the similar material ratio of rock samples, more cataclastic rock mass model samples were developed by manual pouring. The 3D printed structural surface network model was placed into a cube model box with dimensions of 50 mm × 50 mm × 50 mm for pouring. After repeated tests, it was found that after 1/3 of the cement slurry was injected into the model box within which the 3D printed structural surface network model was placed, with pouring continued thereafter, the obtained samples demonstrated the highest integrity. After a sample was poured, it was demoulded after 3 days of air drying in ambient air and cured for 28 days. The final pouring sample is shown in Fig. 6. To simplify the following description, the cataclastic rock mass samples containing 30°, 60°, and 90° intersecting joint network models were denoted as the Type-1, Type-2, and Type-3 samples, respectively.

Five specimens of the Type-1, Type-2, and Type-3 samples were selected for sonic wave testing, and the test results are shown in Table 2. Overall, the sample sonic wave velocity values for each type of specimen were significantly close, and the poured samples exhibited a high degree of homogeneity. In the sonic wave test, with an increase in the development of joints in the rock masses, the wave velocity and dynamic elastic modulus decreased. The wave speed test results revealed that the average wave speeds of the Type-1, Type-2, and Type-3 samples were 3039.48 m/s, 2947.11 m/s, and 2893.48 m/s, respectively. Moreover, the calculated average dynamic elastic moduli were 17,540.10 MPa, 15,285.83 MPa, and 14,630.52 MPa, respectively. The cataclastic degree of the Type-1 sample was the smallest, followed by the Type-2 sample; and that of the Type-3 sample was largest.

**Table 2:** Sonic wave velocity test results.

Sample No	Intersection angle of joints (°)	Sonic wave velocity (m/s)	Average sonic wave velocity (m/s)	Unit weight (g/cm^3^)	Dynamic modulus of elasticity (MPa)	Average Dynamic modulus of elasticity (MPa)
01	30	3039.48	3039.48	1.87	17,895.19	17,540.1
02		3131.4		1.87	17,986.94	
03		2992.77		1.87	18,107.07	
04		3092.72		1.87	17,886.39	
05		3054.99		1.87	17,797.1	
06	60	2947.11	2947.11	1.87	15,158.29	15,285.83
07		2814.59		1.87	15,452.31	
08		2911.93		1.87	15,445.22	
09		2864.81		1.87	14,590.72	
10		2752.7		1.87	15,217.87	
11	90	2893.48	2893.48	1.87	13,566.54	14,630.52
12		2793.48		1.87	13,482.15	
13		2853.48		1.87	12,602.04	
14		2655.97		1.87	13,279.27	
15		2783.3		1.87	13,863.2	

**Figure 7:**
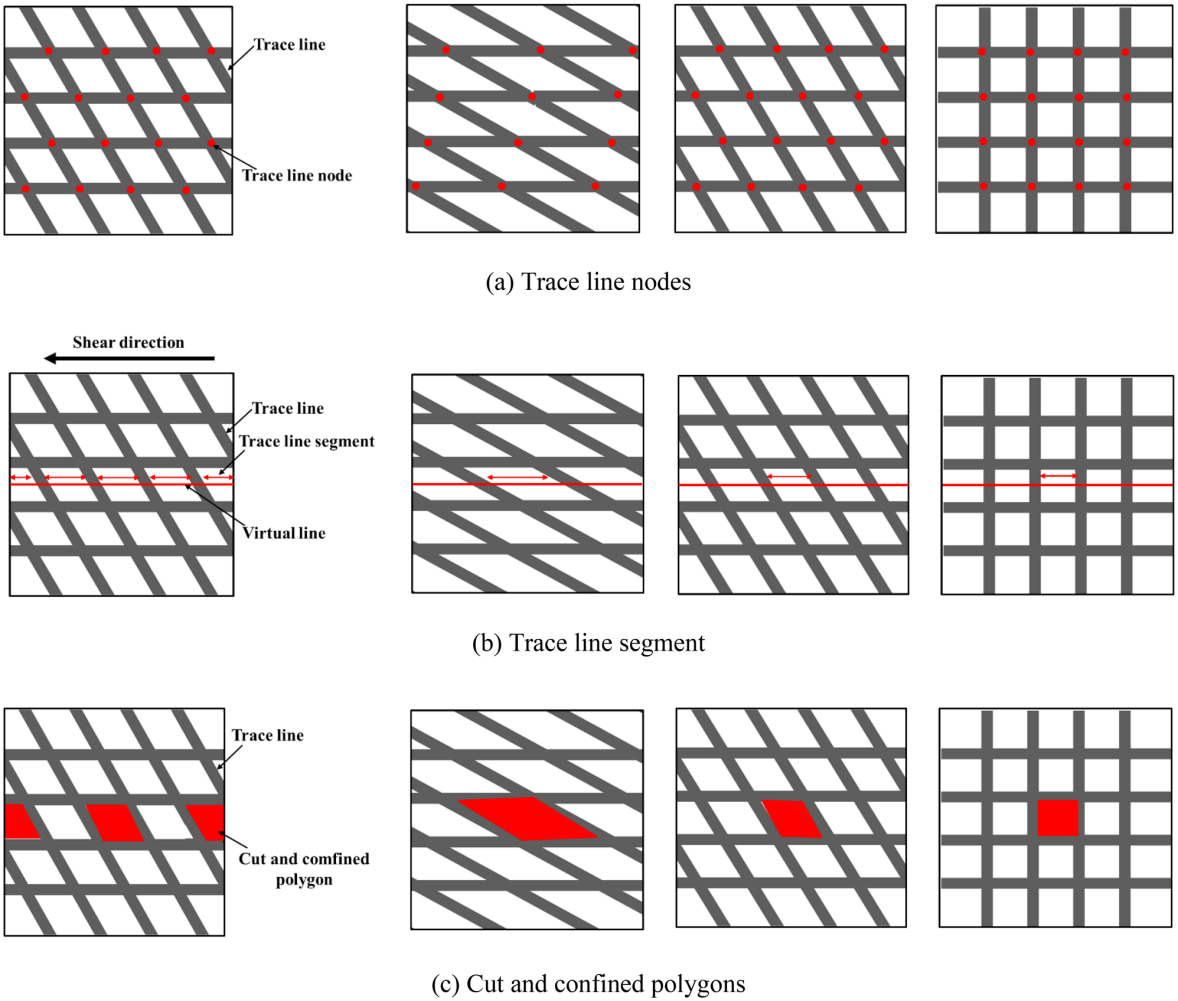
Quantitative indicators of structural characteristics of cataclastic rock mass.

Considering that the cataclastic rock mass samples designed in this experiment were all composed of regularly distributed joints, and the random distribution of the joints was not considered; the trace line nodes index, trace line segment length, and block area that was cut and confined by the trace line were used to evaluate the fragmentation degree of the experimental samples. The trace line nodes index (TLNI) was proposed by Dong et al.^1^, and is defined as the number of trace line nodes on a unit trace line in the unit trace line plane area (Fig. 7a):

$$TLNI=\frac{{N}_{0}}{{N}_{L}}$$

where ${N}_{0}$ is the number of trace line nodes in the unit trace line plane area, and ${N}_{L}$ is the number of trace lines in the unit trace line plane area. This equation indicates the ratio between the number of trace line nodes and the number of trace lines, in addition to the degree of overlap between the trace lines in the trace line nodes. As TLNI increases, the number of parts that overlap in the trace line plane increases, in addition to the cataclastic the rock mass.

The trace line segment length is defined as the line segment length on the trace line that can be measured by arranging a virtual measuring line along the shear direction from the midpoint of the sample, and with a decrease in the length of the segment, the fragmentation of the cataclastic rock mass is more significant (Fig. 7b). Furthermore, the cataclastic texture rock mass is confined by the trace lines, thus leading to the formation of multiple irregular polygons in the trace line plane, which are referred to as crack surface polygons (Fig. 7c). With a decrease in the area of the polygon, the cataclastic rock mass is more fragmented.

Based on calculations, the trace line nodes index of the Type-1, Type-2, and Type-3 samples were 1.2, 1.6, and 2.0, respectively; the average trace line segment lengths of the blocks that were cut and confined by the trace lines were 2 cm, 1.15 cm, 1 cm, respectively; and the areas of the crack surface polygons were 2 cm^2^, 1.15 cm^2^, and 1 cm^2^. The cataclastic degree of the Type-1 sample was the smallest, followed by the Type-2 sample; and that of the Type-3 sample was the largest.

### Direct shear test

**Figure 8:**
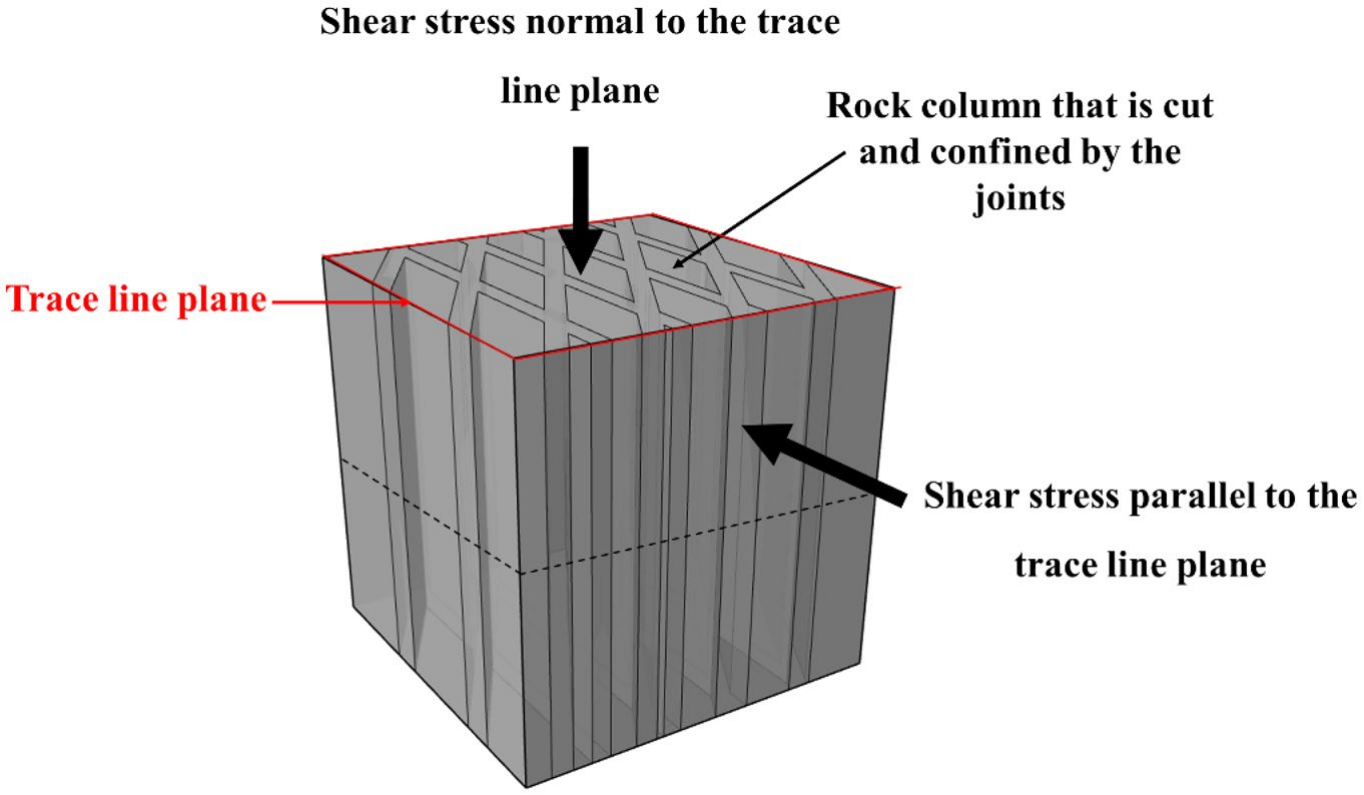
Schematic diagram of test shear direction.

A portable and multi-functional test device for rock mechanical performance, which was independently developed by Chengdu University of Technology, was used to perform physical direct shear tests^20^. The shear tests were carried out under the normal stress was 0.5 MPa, 1.0 MPa, 1.5 MPa, and 2.0 MPa, respectively. The intersection plane of the joint was defined as the trace line plane (Fig. 8). The design scheme of the direct shear test included the shear stress parallel to the plane of the trace line plane, and the shear stress normal to the trace line plane (Fig. 8). During shearing, the normal stress was first applied at a constant value, and then the shear load was gradually applied. Simultaneously, the shear and normal displacement under each level of shear load were measured and recorded.

## Results and discussion

### Analysis of shear failure phenomenon (for shear stress parallel to joint plane)

#### Upper block

**Figure 9:**
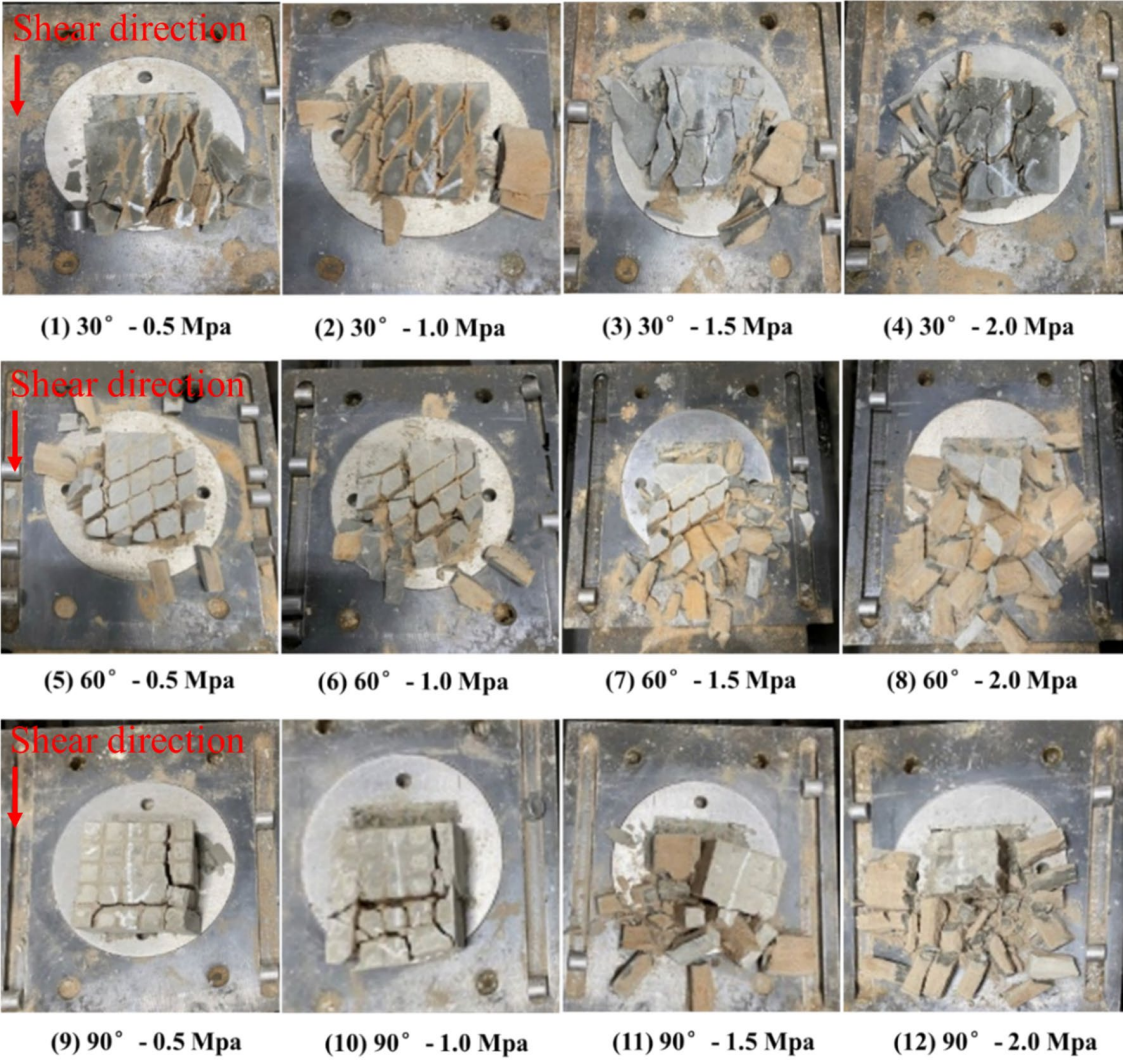
Shear failure phenomenon of the upper blocks of the samples.

Based on the test results, under different normal stresses, the upper halves of different samples exhibited different degrees of relaxation and fragmentation along the shear failure plane (Fig. 9). Based on the failure phenomenon of the upper block after shearing, the failure phenomenon of the upper block was divided into two failure modes: (1) vertical splitting failure along the joint, i.e., the upper block cracked along the columnar joint surface; and (2) rock column splitting failure, i.e., the rock column split along the joint and rock column. Both failure modes were accompanied by significant volume-expanding phenomena, and the main difference was with respect to whether or not the rock column underwent fracturing damage. As observed from the shear failure phenomenon of the upper block, when the normal stress was 0.5 MPa, the failure of the rock mass under the normal stress was vertical splitting failure along the joint; and when the normal stress exceeded 0.5 MPa, the rock column splitting failure was gradually promoted.

In particular, the fracture of rock column is a characteristic of brittle material failure. He et al.^21^ verified the extreme value of the minor tension stress at the crack tip under normal compressive load based on Griffith theory, which revealed that splitting failure occurs during the compression process corresponding to the gradual characteristics of rock failure. Furthermore, the criterion of the occurrence of rock splitting is as follows: the value of the longitudinal load should be high, and the value of lateral stress should be low or zero. In addition, there should be short-term application of loads or no subsequent loads. The theory proposed by He et al.^21^ can explain the phenomenon observed in this test. By analysing the shear pattern after the shear test, it was found that the splitting failure of the rock column mainly occurred along the vertical direction, which demonstrated that the splitting failure of the rock column was mainly formed in the application of the initial normal stress. In the shear test, during the initial normal load application process, the side pressure of each rock mass sample was almost zero. In particular, under short-term normal load application, the rock column underwent splitting failure, and with an increase in the normal load, the impact increased, and the splitting failure of the rock column was more significant.

#### Shear failure plane

**Figure 10:**
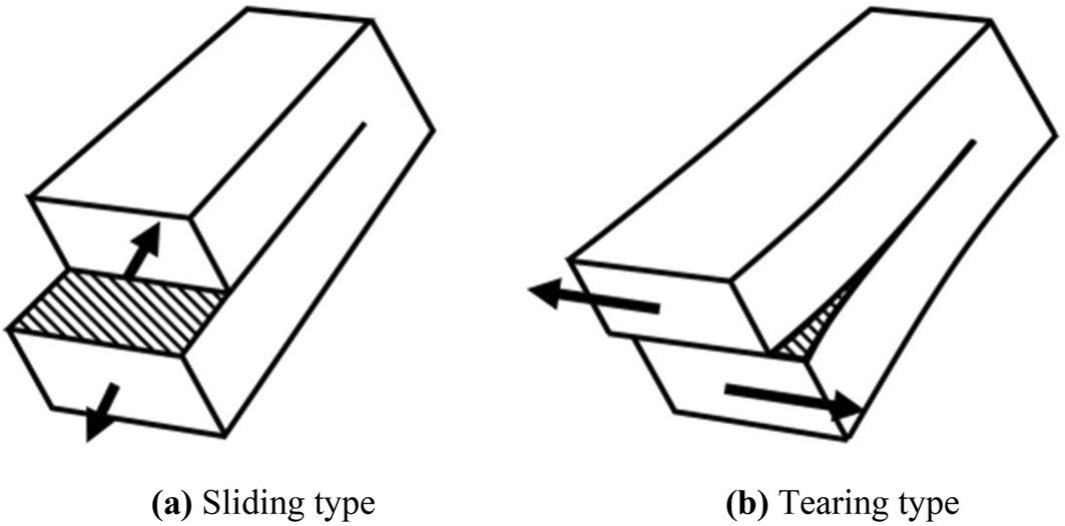
Schematic diagram of shear crack.

Irwin^22^ classified two types of shear cracks: (1) sliding type, where the displacement of particles is parallel to the crack surface and perpendicular to the crack front (Fig. 10a); and (2) tearing type, where the particle displacement is parallel to the crack surface and crack front (Fig. 10b). Hence, Irwin^22^ proposed the shear crack intensity factor, which can be expressed as follows:

1 $${K}_{J}=Y\sigma \sqrt{c}$$

where ${K}_{J}$ is the stress intensity factor; Y is the quantity related to the crack shape and loading method for the centre of the infinite body through the crack, where $Y=\sqrt{\pi }$; $\sigma$ is the applied stress; and c is the crack length. According to Eq. (1), during the shearing process of the rock mass (before the peak strength), the shear stress continuously increases, and the stress intensity factor ${(K}_{J})$ at the crack tip increases continuously. When the stress intensity factor ${(K}_{J})$ increases to a certain critical value $({K}_{Jc})$, the stress in a certain area of the crack tip can be sufficiently large to damage the material, thus resulting in the unstable expansion of the crack and the fracture of the material.

**Figure 11:**
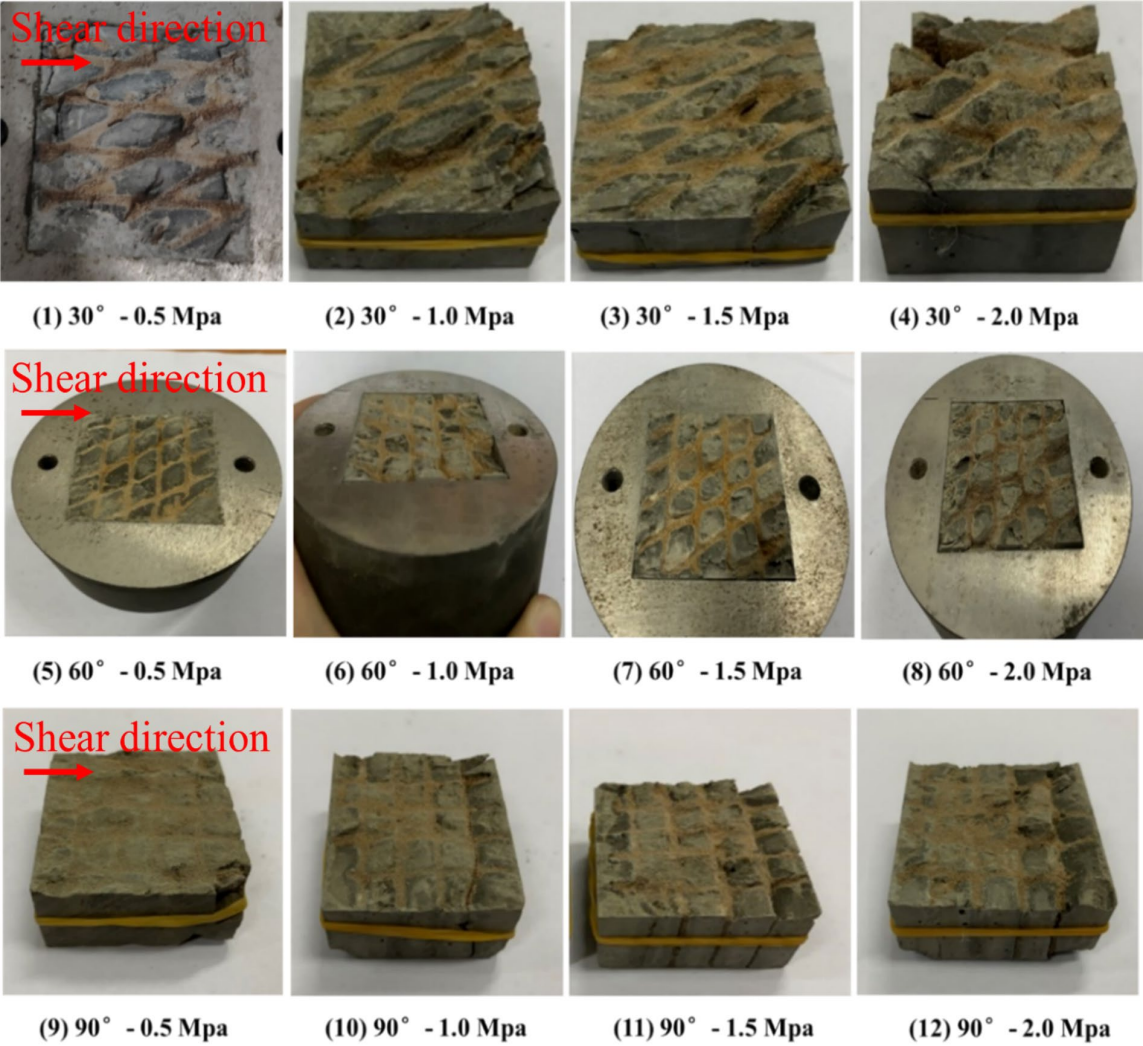
Shear failure phenomenon of the shear failure plane.

The shape of the shear failure plane of each sample is shown in Fig. 11. Under a low normal stress and at the initial stage of high normal stress shearing, significant scratches were in the shear failure plane, which means that under a low normal stress and the initial stage of high normal stress shearing the failure mode of the shear failure plane is mainly sliding type. In the middle and late stages of high normal stress shearing, there were no significant shear scratches, and the shear failure plane of the rock column was exhibited as compression–shear-type tearing failure. This was due to the rock mass and rock column splitting failure of the upper rock block under the action of a high normal stress, and the volume-expanding phenomenon was significant. Under the lateral confinement of the shear box, the squeezing and biting forces of the initially fractured rock column gradually increased, which caused the shear stress to increase; thus resulting in the continuous increase of the ${K}_{J}$ at the crack tip in the middle and late stages until it reached ${K}_{Jc}$, and finally the rock column further tearing failure. It should be noted that the undulation and anisotropy of the shear failure plane of the Type-1 sample were significantly different from those of the Type-2 and Type-3 samples, and formed a step-like failure pattern. This may be because the tip effect of the rock column cut by the joint confinement of the Type-1 sample was more significant, and the rock column of the Type-1 sample was more prone to tearing damage during the entire shearing process. The test results revealed that when the rock block that was cut and confined by the trace line exhibited a significant tip and the anisotropy of the rock block was larger, the end stress concentration effect of the cataclastic rock mass was more significant during the shear process, and the failure mechanism was mainly compression shear tear failure, which eventually formed a stepped failure mode.

### Shear stress and shear displacement characteristic curve analysis

Based on the test results, the shear stress–displacement curves of different samples were all peak-type curves that can be classified into three stages, namely, the pre-peak elastic stage, post-peak softening stage, and residual stage. Given that the post-peak curve can indicate the variation trend of the deformation and failure of the shear failure plane to an extent, based on the smoothness of the curve, we divided the curve into three categories as follows. I. The post-peak curve was relatively flat and regular, and the residual stage was relatively stable, thus reflecting that the shear failure plane of the sample after shearing was relatively straight; II. slight fluctuations were observed in the post-peak softening stage or the residual stage, thus reflecting that the shear failure plane after shearing of the sample exhibited slight fluctuations; and III. there were large fluctuations in the post-peak softening stage or residual stage, and a “stress plateau” was observed, thus reflecting that the shear failure plane of the sample after shearing exhibited a greater degree of roughness and fluctuations.

**Figure 12:**
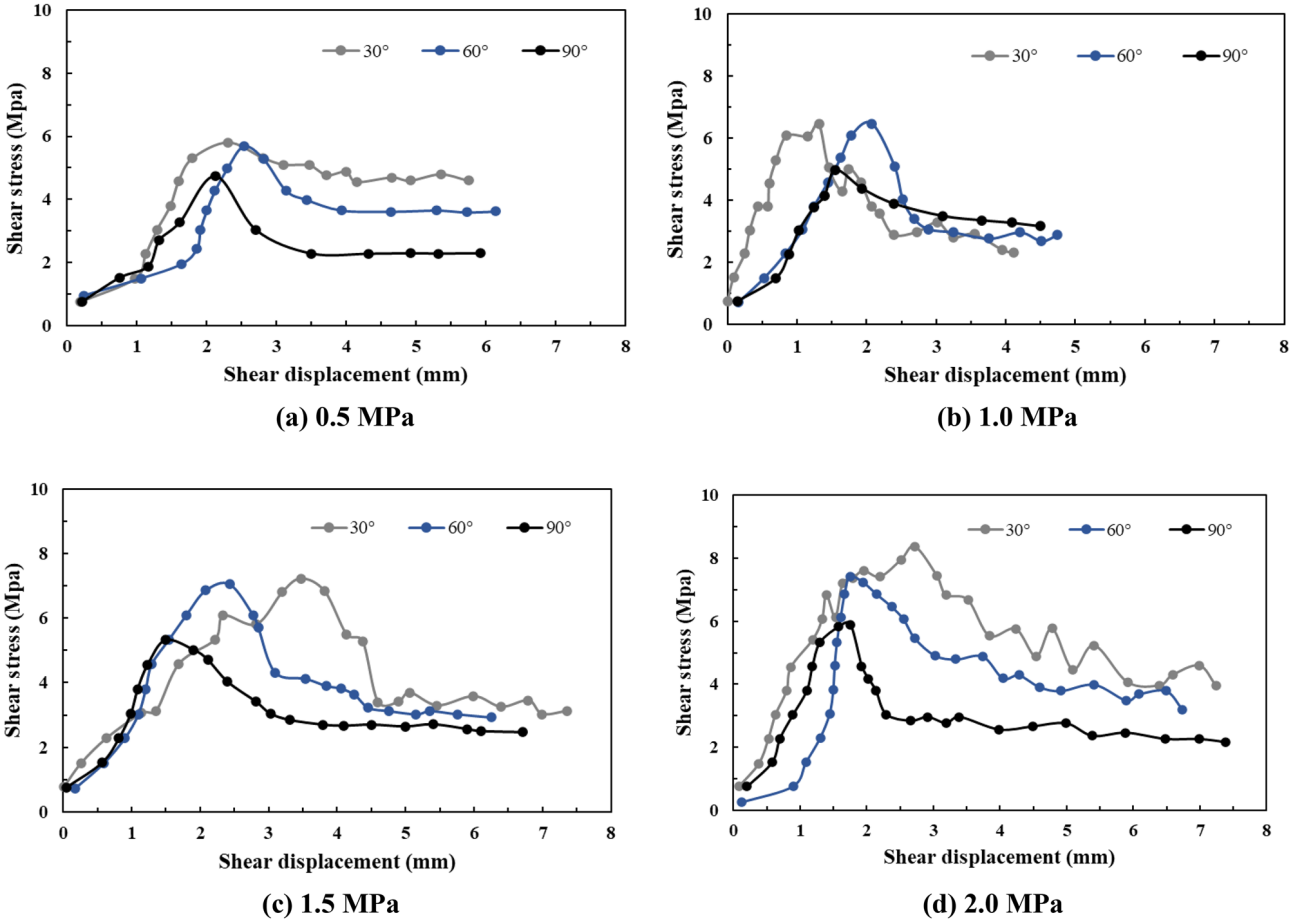
The shear stress–shear displacement curves of different samples.

As can be seen from Fig. 12, with an increase in the normal stress, the shear stress–displacement curves of the different samples all transitioned from Category I to Category III. Under the same normal stress conditions, the shear stress displacement curve of the Type-1 sample was significantly different from that of the Type-1 and Type-2 samples, and the shear stress displacement curve of the Type-2 sample was slightly different from that of the Type-3 sample, which mainly indicates that the fluctuations of the Type-1 sample curve were more significant than those of the Type-2 and Type-3 samples, and the fluctuations of the Type-2 sample curve were more significant than those of the Type-3 sample. This indicates from that the roughness of the shear failure plane of the Type-1 sample was significantly greater than that of the Type-2 and Type-3 samples, and the roughness of the shear failure plane of the Type-2 sample was slightly greater than that of the Type-3 sample.

### Shear stress

As can be seen from Fig. 12, although the failure curves of the Type-2 and Type-3 samples were highly similar, the peak shear strength of the Type-2 sample was closer to that of the Type-1 sample and larger than that of the Type-3 sample. Furthermore, the peak shear strength of the Type-1 sample was slightly larger than that of the Type-2 sample. From the analysis of the cataclastic rock mass samples, the main difference between the Type-1, Type-2, and Type-3 samples was with respect to the different shapes and sizes of the confined and cut blocks in the cataclastic rock mass. Among them, the shapes of the confined and cut blocks of the Type-1 and Type-2 samples were similar, and the block sizes were significantly different. However, the peak strength difference was not significant, which indicates that the size of the confined and cut blocks in the unit area is not the main factor influencing the peak strength of cataclastic rock mass. The sizes of the confined and cut blocks in the Type-2 and Type-3 samples were highly similar; however, the shapes were completely different, and the peak strengths were significantly different, which indicates that the main influencing factor of the cataclastic rock mass strength is the shape of the confined and cut blocks in the cataclastic rock mass per unit area.

**Figure 13:**
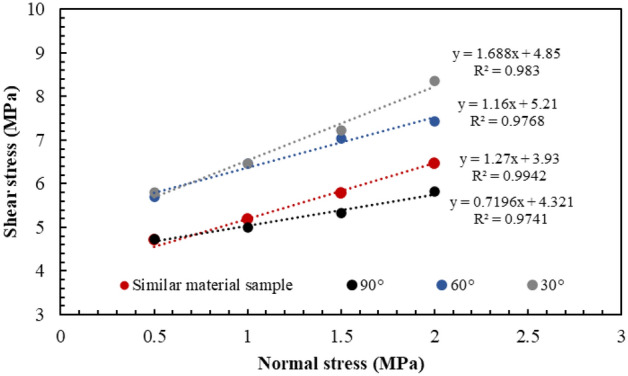
Fitting results of normal–shear stress curves.

Furthermore, based on the analysis of the fitting test results (Fig. 13 and Table 3), the cohesion values of the Type-1, Type-2, and Type-3 samples were 4.85 MPa, 5.42 MPa, and 4.32 MPa, respectively; and the friction angle values were 59.38° and 45.98° and 35.75°, respectively. With an increase in the intersection angle of the joints, the cohesion exhibited an increasing trend followed by a decreasing trend. The internal friction angle exhibited a decreasing trend. In addition, the shear stresses of the Type-1, Type-2, and Type-3 samples were compared with the shear stresses of the pure rock samples. Under the same normal stress conditions, the shear strengths of the complete rock samples were smaller than those of the Type-1 and Type-2 samples, and larger than that of the Type-3 sample. The strength of the cataclastic rock mass was not necessarily lower than that of the intact rock.

**Table 3:** The fitting test results of the cohesion and friction angle.

Sample	C/MPa	Φ/°
Similar material	3.93	51.8
30° (Type-1 sample)	4.85	59.38
60° (Type-2 sample)	5.42	45.98
90° (Type-3 sample)	4.32	35.75

### Shear stress perpendicular to the joint plane

#### Analysis of the shear failure phenomenon

**Figure 14:**
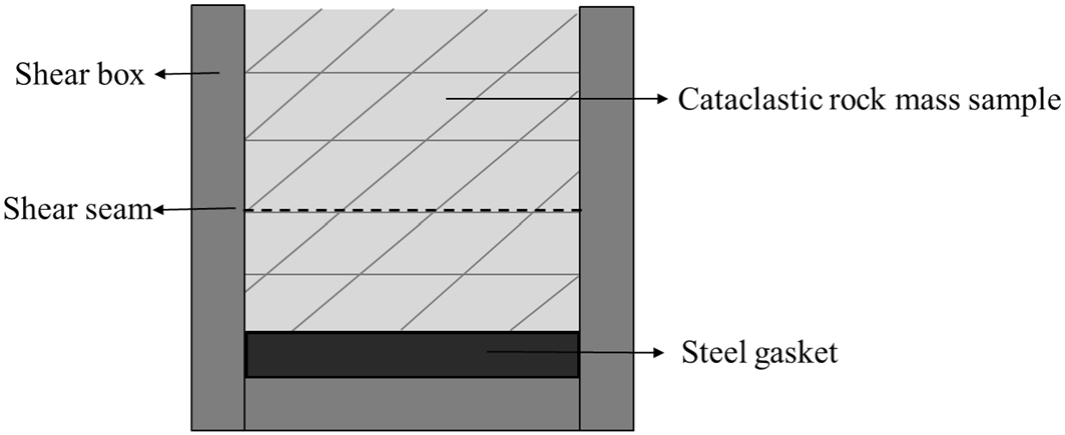
The shear mode of the shear test with shear stress perpendicular to the joint plane.

**Figure 15:**
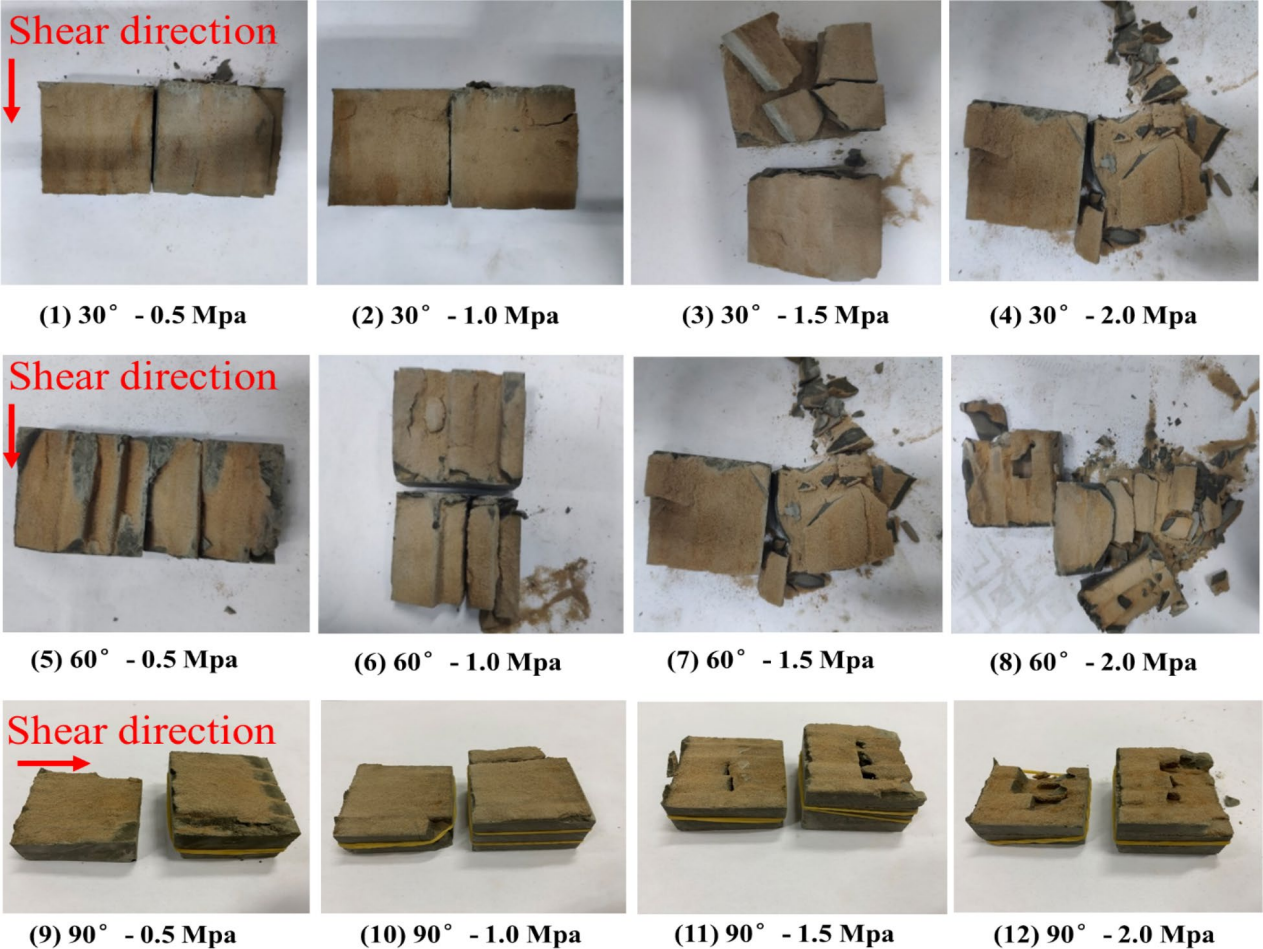
The shear failure planes of the different samples.

**Figure 16:**
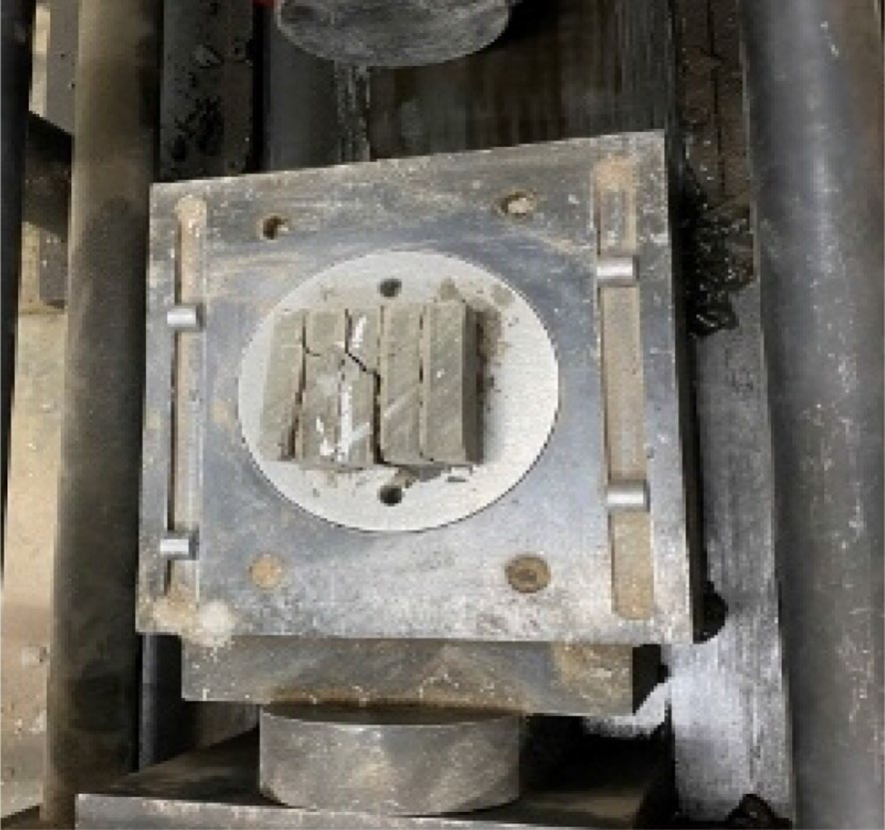
Breakage of the cemented rock column along the joint surface.

**Figure 17:**
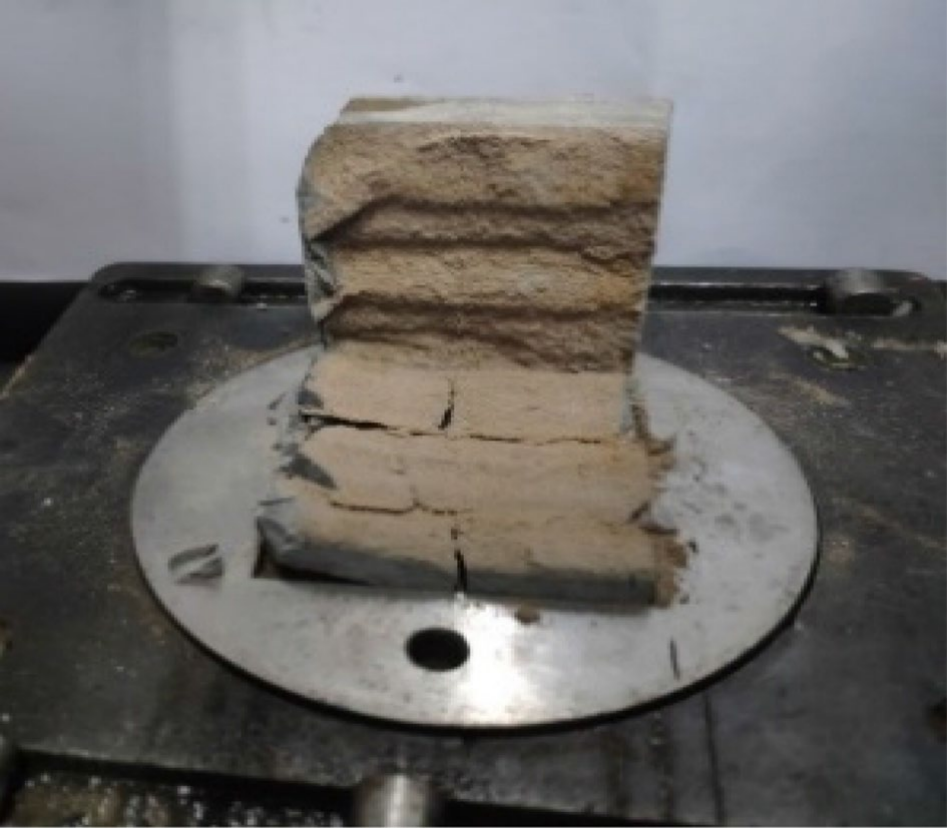
Significant compression failure of the shear failure plane.

The shear mode of the shear test with shear stress perpendicular to the trace line plane is shown in Fig. 14. During the shear test, the joint surface of the sample was placed at the position of the shear seam by positioning a gasket under the sample to ensure that the sample was sheared along the joint surface during the shearing process. The samples after shearing are shown in the Fig. 15, where the upper and lower rock blocks and the shear failure plane exhibited high integrity after shearing under the condition of low normal stress. With an increase in the normal stress, the rock block gradually underwent fragmentation, and the degree of fragmentation of the shear failure surface increased. Based on the shear test results analysis, there were two main failure phenomena; i.e. the upper block of the sample the cemented rock column breaks along the joint surface, and the shear failure plane underwent significant compression shear failure. Images of these two typical phenomena, as observed in the test, are shown in Figs. 16 and 17.

#### Shear stress and shear strain characteristic curve analysis

**Figure 18:**
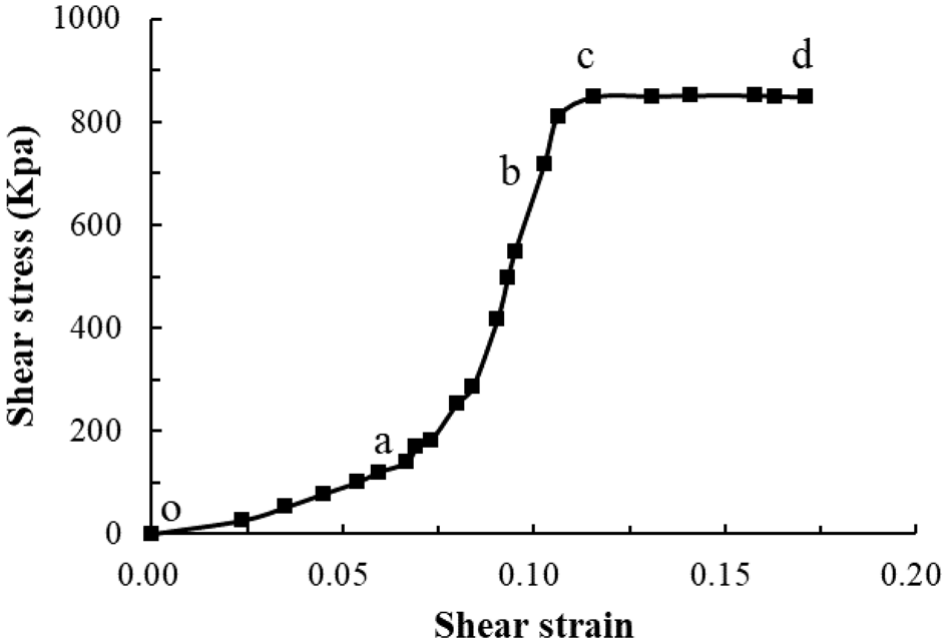
Shear stress characteristic curve of single joint surface for plastic deformation.

In general, the shear stress characteristic curve of the single joint surface of the plastic deformation can be divided into four stages (Fig. 18), as follows. (1) Closure of existing cracks (o–a): in this phase, the density of filler increases, the shear deformation is nonlinear, and the stress–strain curve is concave. (2) Elastic deformation (a–b): the joint of rock mass transitions from a discontinuous medium to a nearly continuous medium, the matrix deforms, and the material exhibits intact rock behaviour characterized by constant stiffness. (3) Fracture development (b–c): inelastic deformation and plastic behaviour can be observed, in which the curve loses its linearity with a significant increase in strain as microfractures propagate in a stable manner. (4) Fracture coalescence (c–d): fracture propagation, coalescence, and interaction induce the degradation of the mechanical properties, in addition to damage to the joint of the rock mass.

**Figure 19:**
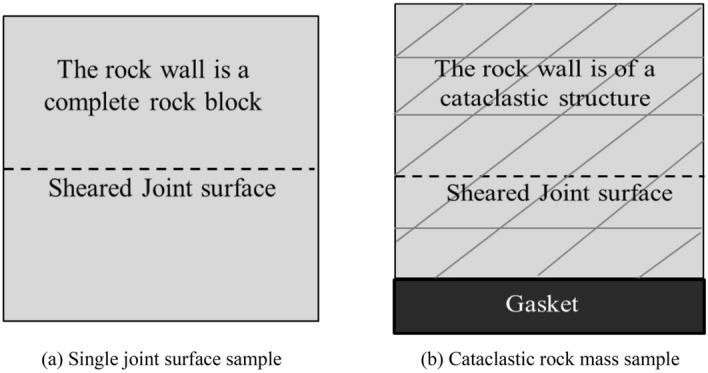
Comparison between the single joint surface rock mass sample and cataclastic rock mass sample.

Based on the test results, under the action of a low normal stress, the stress–strain curve obtained from the test was highly similar to the brittle deformation shear stress–strain characteristic curve. Under the action of a high normal stress, there was almost no closure stage of existing cracks, and an almost direct transition to the elastic stage. This may be because the cataclastic rock mass sample in the test was different from the conventional single joint surface sample. The rock wall of the single joint surface sample was complete (Fig. 19a), and the rock wall had a slight influence on the deformation and damage of the joint plane in the shear process. In the test, the rock wall of the sample was controlled by the joints, and the structure of the rock wall could be considered as the cataclastic rock mass (Fig. 19b). In the above damage phenomenon analysis, under a higher normal stress, during normal stress application, the cemented rock column underwent breakage along the joint surface, which led to the cracking of the rock wall structural bulk. Thus, the closing stage of the rock mass in the shear process was not significant. This indicates that in the initial stage of the joint surface shearing of the cataclastic rock mass, the compaction deformation under low normal stress conditions is dominated by the closure of shear joint planes; and in the initial stage of deformation under high stress conditions, the deformation of the rock mass structure is dominant.

**Figure 20:**
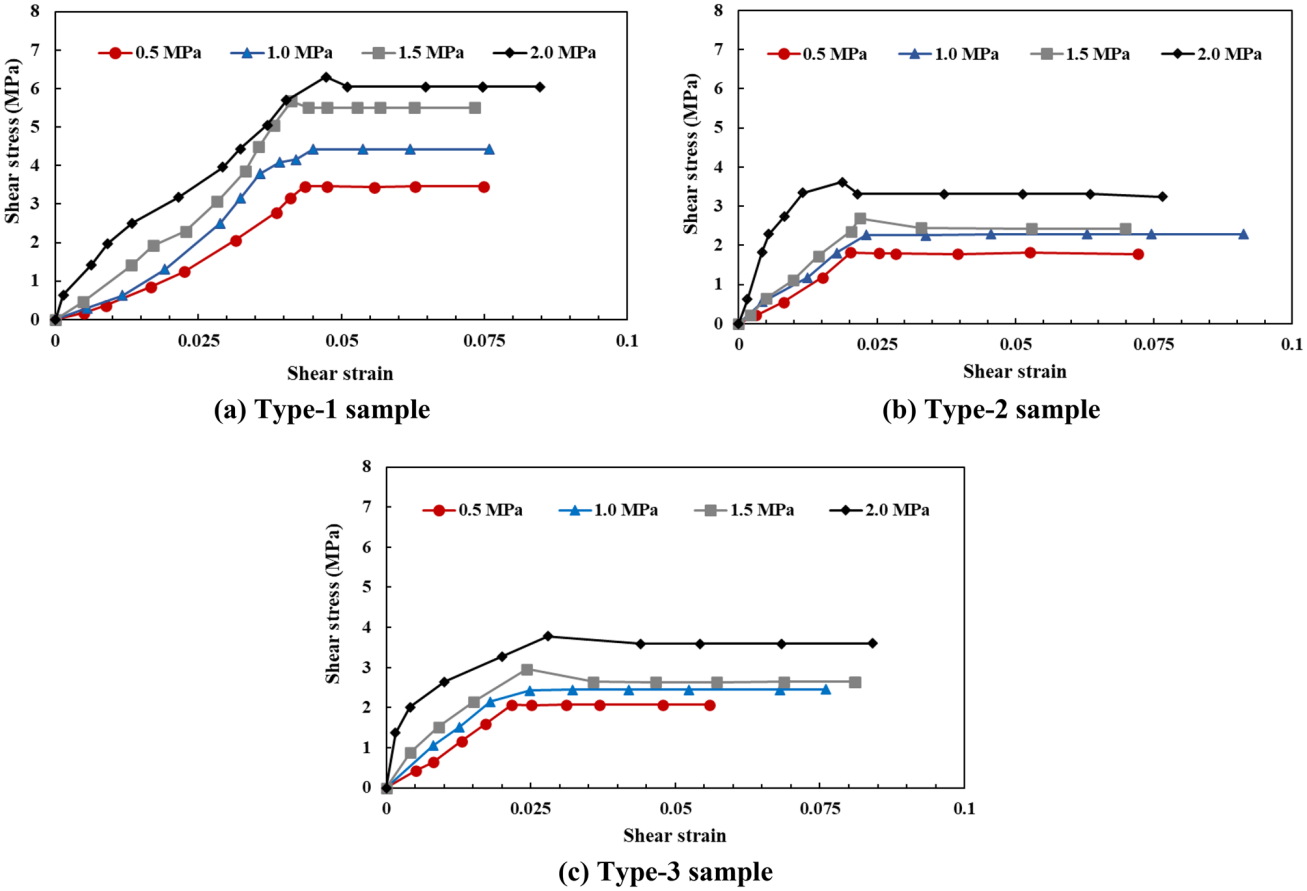
The shear stress–shear strain curves of different samples.

Most of the shear stress–shear strain curves exhibited local peaks under the high normal stress conditions (1.5 MPa and 2.0 MPa, as shown in Fig. 20). During the application of normal stress, the cemented rock column underwent breakage along the joint surface of the rock wall, thus resulting in dilatancy. The shear failure plane was subjected to significantly high pressures and sheared during the shear process, thus resulting in the shear failure surface accompanied by the shear failure of local rocks. Thereafter, overall sliding failure occurred. This indicates that during the shear process, the plastic deformation failure of the shear failure plane was the main process, accompanied by local brittle deformation failure. Moreover, the structural characteristics of the rock wall had a significant influence on the deformation and failure process of the shear failure plane of the rock mass.

#### Shear stress

**Figure 21:**
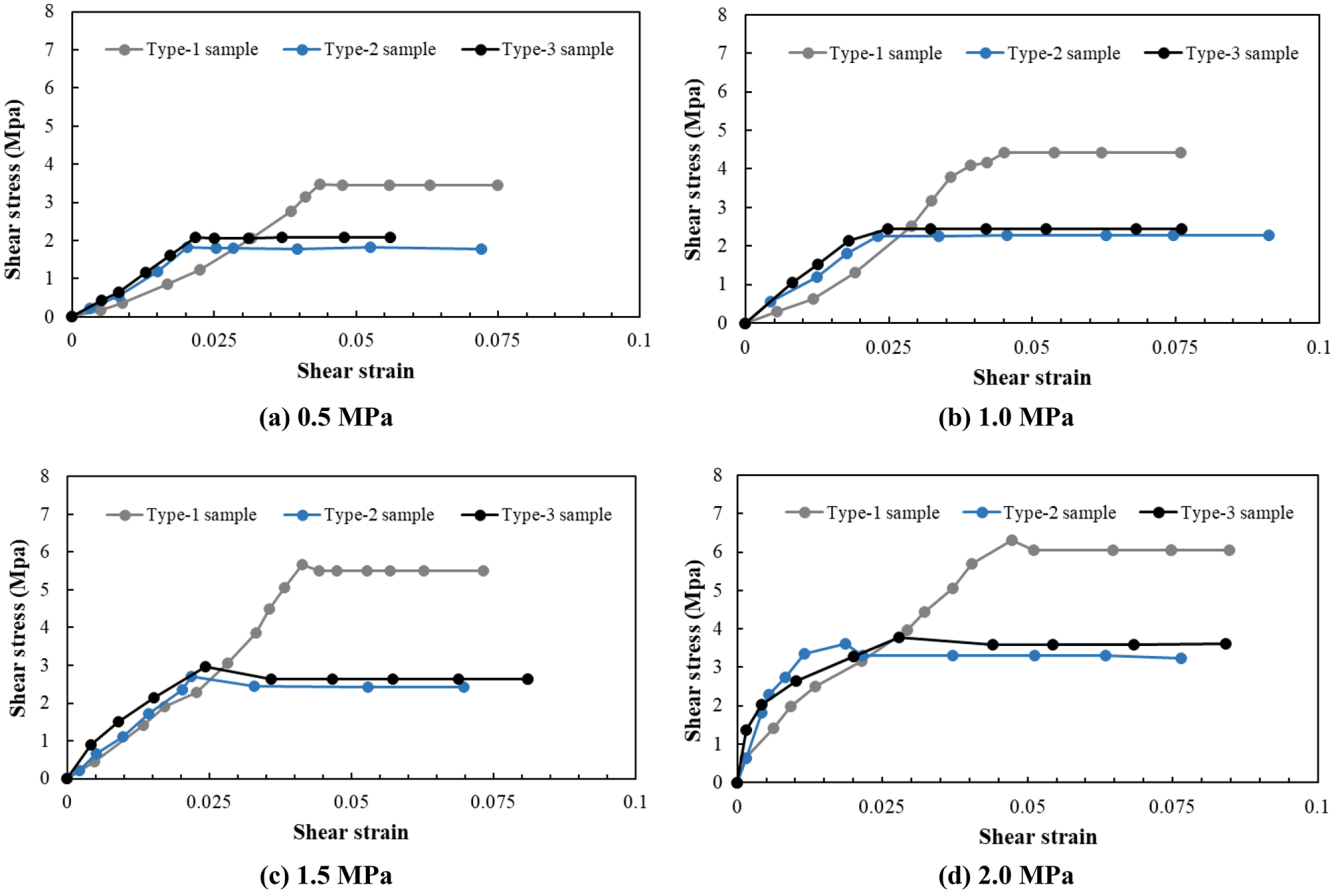
The shear stress–shear strain curves of different samples.

**Figure 22:**
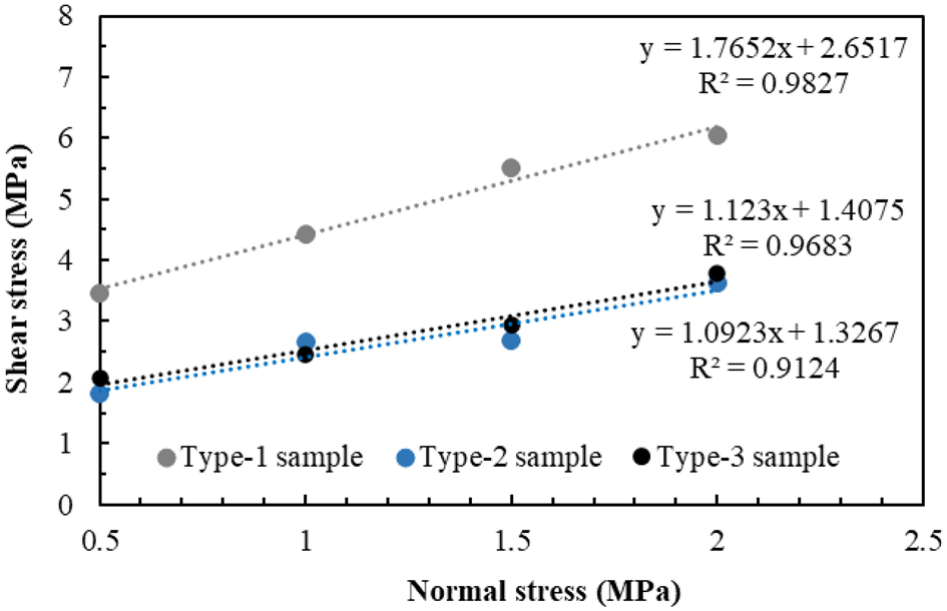
Fitting results of the normal stress–shear stress curves.

Based on the test results, under the same normal stress conditions, the shear strength of the Type-1 sample was significantly larger than that of the Type-2 and Type-3 samples (Fig. 21). Based on the analysis of the damage phenomenon, under the same normal stress conditions, the integrity of the rock wall of the Type-1 sample was superior, and the shear failure plane was relatively complete. In particular, the contact area of the shear failure plane was the largest during the shearing process, and the sliding resistance of the joint was large. For the Type-2 and Type-3 sample, the cemented rock column underwent breakage along the joint surface of the rock wall during the application of normal stress. Thereafter, with an increase in shear displacement, the extrusion deformation of the rock wall damaged by fracturing induced further fragmentation, thus resulting in the structural relaxation of the rock wall and decrease in the shear stress. Given that the shear strength of the Type-2 sample was lower than those of the Type-3 sample, the degree of fragmentation of the rock wall of the Type-2 sample was greater than that of the Type-3 sample, and the strength deterioration was more significant. The fitting results of the normal stress–shear stress curve are shown in Fig. 22. The cohesion values of the Type-1, Type-2, and Type-3 samples were 2.65 MPa, 1.40 MPa, and 1.32 MPa, respectively; and the friction angles were 60.5°, 48.4°, and 47.5°, respectively. The cohesion and the internal friction angle decreased with an increase in the joint intersection angle. Due to the few types of intersection angles of joints considered in this test, this rule can only be verified by conducting further tests.

For a comparative analysis, a direct shear test was conducted on 3D printed intact samples with printing materials and printing methods consistent with the joint network model, and the test process was the same as previously mentioned. The test results of the sandstone samples were approximately equivalent to the shear strength of the structural plane of the complete rock wall sample. Based on the test results, the cohesion of intact sample was 2.28 MPa, and the internal friction angle was 37.2°. Furthermore, based on the fitting results of cataclastic rock samples and intact samples, the shear strengths of shear structural planes under different normal stresses were calculated, and the results are shown in Table 4. Irrespective of the normal stress, the shear strength of the Type-1 sample was greater than that of the intact sample; and with an increase in normal stress, the shear strengths of the Type-2 and Type-3 samples gradually increased until they exceeded that of the intact sample. Hence, in addition to the normal stress and roughness of the joint surface, as proposed in previous studies, the structure of the rock wall and the deformation characteristics in the shear process should be considered when calculate the shear strength of the rock mass.

**Table 4:** The shear strength of different sample types under different normal stress conditions.

Sample type	Normal stress/MPa							
	0.5	1	1.5	2.0	2.5	3.0	3.5	4
Intact sample	2.66	3.04	3.42	3.80	4.18	4.56	4.94	5.32
Type-1 sample	3.53	4.42	5.30	6.18	7.06	7.95	8.83	9.71
Type-2 sample	1.87	2.42	2.97	3.51	4.06	4.60	5.15	5.70
Type-3 sample	1.97	2.53	3.09	3.65	4.22	4.78	5.34	5.90

## Conclusions

Three-dimensional printing technology is an effective tool for the investigation of the physical and mechanical properties of cataclastic rock masses, as it transforms digital simulation results into lab-testable rock mass models that facilitate traditional rock mass analysis. The workflow presented in this paper can be readily modified to meet the requirements of other cataclastic rock mass joint network geometries, and more sophisticated algorithms can be incorporated for mechanical analysis. With the advancement of 3D printing technology and materials, cataclastic rock mass models at the original scale can be developed in the near future. The conclusions of this study are presented below:

1. When the shear stress is parallel to the trace line plane, and when the rock block that is cut and confined by the trace line exhibits a significant tip, the end stress concentration effect of the cataclastic rock mass was more significant during the shear process with the anisotropy of the rock block increased. And the failure mechanism of the rock column is mainly compression shear tear failure, which leads to the formation of a stepped failure mode.
2. When the shear stress is parallel to the joint plane, the shape of the confined and cut rock blocks is the main factor influencing the strength of the cataclastic rock mass. When only two groups of intersecting joints are considered, with an increase in the intersection angle of the joints, the cohesion exhibits an initial increasing trend, followed by a decrease; and the internal friction angle exhibits a decreasing trend. Moreover, the strength of cataclastic rock mass is not lower than that of intact rock in all cases.
3. When the shear stress is perpendicular to the trace line plane, the structure of the rock wall has a direct influence on the deformation and failure process of the shear failure plane and the shear strength. In the initial stage of the joint surface shearing of a cataclastic rock mass, the compaction deformation under low normal stress conditions is dominated by the closure of shear failure planes; and in the initial stage of deformation under high stress conditions, the deformation of the rock mass structure is dominant.
4. The physical and mechanical properties of the shear failure plane of a cataclastic rock mass are closely related to the joint–rock wall system characteristics. When considering the calculation or value of the shear strength of cataclastic rock mass under actual conditions, the shape and combination form of the confined cutting rock block should be comprehensively considered, in addition to the shear direction, structural characteristics of the rock wall, and failure of the rock wall during the shear process.

## Data Availability

The datasets used and/or analysed during the current study available from the corresponding author on reasonable request.
